# Mental health care in Eastern Europe and Central Asia: An analysis of needs and a call for greater investment

**DOI:** 10.1016/j.lanepe.2021.100182

**Published:** 2021-07-29

**Authors:** Kimberly Hook, Sergiy Bogdanov

**Affiliations:** 1 Boston Medical Center, Department of Psychiatry, Boston, MA, USA; 2 Boston University School of Medicine, Department of Psychiatry, Boston, MA, USA; 3 Massachusetts General Hospital, Department of Psychiatry, Boston, MA, USA; 4 National University of Kyiv-Mohyla Academy, Center for Mental Health and Psychosocial Support, Kyiv, Ukraine

**Keywords:** Global mental health, Eastern Europe, Central Asia, Ukraine, capacity building, mental health

## Abstract

Over the past decade, there has been increased attention to global mental health, which emphasizes improving access to quality mental health care in order to reduce the worldwide treatment gap. However, Eastern European and Central Asian countries and their specific mental health needs have largely been under-emphasized, evidenced by a dearth in literature and funding. Here, we provide an overview of the mental health needs in Ukraine and its challenges with quality care provision as a key example in highlighting these gaps, then broaden our discussion to include parallels with other countries in the Eastern European and Central Asian region. We describe the unique strengths relative to mental health care provision that are present in Eastern Europe and Central Asia and suggest the importance of post-graduate training, regional collaboration, and capacity building specific to research management as strategies to address the current challenges. We stress that greater investment from funders, government, and the global mental health community are needed to improve the current mental health situation in Ukraine, specifically, and Eastern Europe and Central Asia, broadly. We argue that greater attention to Eastern Europe and Central Asia is needed to fully advance the agenda of the global mental health field.

Mental illnesses account for over 30% of years lived with disability worldwide [1], though psychiatric treatment in low- and middle-income countries (LMICs) is often limited [2]. Over the past 20 years, focus on the field of global mental health, and its efforts to reduce mental health disparities, has substantially increased [2]. Despite significant rates of mental and substance use disorders in Eastern Europe and Central Asia, there has been a lack of attention to the implementation of quality, contemporary mental health care services and systems reform throughout the region [3,4].

For this Viewpoint, we specifically highlight the following Eastern European and Central Asian countries: Armenia, Azerbaijan, Belarus, Estonia, Georgia, Kazakhstan, Kyrgyzstan, Latvia, Lithuania, Moldova, the Russian Federation, Tajikistan, Turkmenistan, Ukraine, and Uzbekistan [5]. While we recognize the uniqueness of each country regarding mental health care and reform, we also note the legacy of the Soviet healthcare system, including the continuing impacts of its centrally planned, Semashko-style structure [6] and the historical use of psychiatry as a political tool, [7] which today continues to challenge mental health care provision in primary or community care settings and worsens mental health stigma [8], [9], [10], [11], [12], [13] Echoing existing calls [3], we urge greater awareness of the unique mental health needs of the region. Using Ukraine as a key example, we provide a contextual analysis and highlight the current gaps in mental health training and research. We conclude by drawing parallels with neighboring countries and discussing recommendations for engagement from the global mental health community and associated stakeholders.

## Ukraine: Mental Health Needs and Context

In Ukraine, a lower-middle income country, approximately 33% of the population experiences mental illness in their lifetime [7,14]. Prevalence rates of common mental health disorders, particularly depression, are substantial [7,15], and Ukraine has one of the highest suicide rates in the world [7]. Mental health and substance use disorders are frequently co-occurring, and rates of harmful substance use are identified as critical public health issues that result in significant injury and death [7]. Reports suggest that 40% of deaths among males and 22% of deaths among females between the ages of 20 and 64 are attributable to the effects of alcohol [16].

A variety of public health factors, many of which are equally relevant across the region, interact with and drive these rates of mental and substance use disorders. Specifically, a syndemic of tuberculosis (TB) and HIV has resulted in complex, intersecting comorbidities and challenges. Newly diagnosed cases of HIV from Ukraine and Russia accounted for 75% of all cases in the World Health Organization (WHO) European Region [17], and Ukraine has the second largest burden of TB among European countries [18]. Rates of mental illness are frequently higher among individuals with HIV and/or TB diagnoses, and bidirectional, synergistic impacts between HIV, TB, and mental illness are known to exacerbate one another [19,20]. There is a unique need to ensure that mental health care and substance use treatment become an integral part of the country's responses to HIV and TB, both to treat existing psychiatric conditions and limit onset of mental illness.

In addition to the mental health needs of the general population, the longstanding military conflict in Eastern Ukraine is associated with an increasing number of psychiatric symptoms among local residents. As a result of the conflict, approximately 1.5 million Ukrainians are now identified as internally displaced persons (IDPs), and millions of people are at ongoing risk of losing access to social and health services [21]. A recent study among Ukrainian IDPs indicated that posttraumatic stress disorder prevalence was 32%, depression prevalence was 22%, and anxiety prevalence was 17% [22]. However, 74% of IDPs who likely required mental health care did not receive it, with barriers to care often due to lack of knowledge of where to receive help, poor understanding by health care providers, poor quality of services, and stigma [23]. High rates of alcohol use among this population are identified as another barrier to accessing mental health care [23].

Social factors, including stigma and human rights violations of individuals with psychiatric and substance use disorders, also merit attention. Stigma, including mental health-related stigma, is a social determinant of health that negatively impacts outcomes and perpetuates health disparities [24], [25], [26]. In Ukraine, there is an overwhelming lack of knowledge of mental illness and of effective mental health treatments [27]. This knowledge gap in turn impacts mental health-related stigma, including beliefs that seeking help may be a sign of weakness [28], that it may be better to avoid someone with mental illness [28], and that delivering mental health services in the community may pose a security risk [27]. Other work from Ukraine has identified that mental health-related stigma is associated with delayed treatment seeking and social distancing [29], though a recent study documented improving compassion and concern for individuals with mental illness [27]. In addition to stigma, human rights violations and limited participation of service users in planning and evaluating services are related areas of concern [6,7,30].

As highlighted above, there are significant mental health needs in Ukraine that will likely only become more urgent over the next years and may be worsened by complex social factors. The ongoing COVID-19 pandemic may also coincide with greater mental health needs among the Ukrainian population, as similarly noted in reports from countries worldwide [30,31]. Unfortunately, this will likely only continue to overburden the mental health care system; today, the majority of individuals who need mental health care do not receive it. Less than 5% individuals with a psychiatric disorder access treatment [15], and only 25% of individuals who endorse suicidal thoughts receive help from a professional [14].

### Challenges to Accessing Quality Mental Health Care

#### Mental Health Care System: Past and Present

Ukraine continues to heavily rely on institutionalization as the main form of mental health treatment [32]. This results in 89% of mental health financing used for inpatient psychiatric care, which decreases availability and accessibility of mental health services and is against international recommendations. While efforts are underway to move towards deinstitutionalization, recent changes have resulted in a 50% decrease in budgets for state psychiatric hospitals, which is in turn associated with a diminished mental health workforce [30]. As a compounding concern, of the overall health care budget, only 2.5% is allocated for mental health services, which is suggestive of the lack of esteem given to mental health care [7]. Unsurprisingly, other reports note that allocation of financial resources for mental health care is significantly below the average of the European Union [4].

Regarding treatment for substance use disorders, providers work in silos, such that psychiatrists treat psychiatric conditions, and narcologists treat alcohol and drug use disorders, without integration in care [7]. Concurrent treatment for co-occurring disorders is typically not available in Ukraine, though is recommended as best practice [33,34]. Among state-funded substance use treatment facilities, a medical model of treatment (i.e., detoxification, drug replacement therapy) is emphasized, and non-pharmacological treatments are seldom available [35].

Fortunately, some signs of change are on the horizon. Among other health system reforms, there are efforts to modernize the mental health care system in Ukraine. While Ukraine has not adopted a national mental health action plan, a concept note passed in 2017 outlines suggested updates and recommendations [36], including a transition to increased access to community-based care [6]. Ukraine is also participating in the WHO Special Initiative for Mental Health (2019-2023): Universal Health Coverage for Mental Health, which aims to support Ukraine as it increases the quality and accessibility of mental health services. Nevertheless, a challenge to sustained momentum is the frequent change in political leaders and priorities over the past years [37]; one clear example is the four Health Ministers appointed over the course of the last year alone [38].

#### Gaps in Mental Health Training and Research

Beyond challenges within the mental health care system, there are related factors that must be strengthened to improve issues related to provision of quality, evidence-based mental health interventions. Increasing the quantity of providers and services without equal attention to strengthening mental health education will limit the effects of any systems change. As the current mental health care is generally recognized to be of low quality [7], this is of critical importance.

Formal psychological education in Ukraine lacks training in evidence-based treatment approaches, and professional licensure of mental health professions does not exist [7]. Instead, psychologists are more frequently trained as pedagogical workers and prepared to work in school settings [35]. The only opportunities to obtain training in evidence-based practices, such as cognitive behavioral therapy, come through private universities or courses [7]. Psychologists (i.e., those with a bachelor or masters degree) commonly do not have formalized experience providing talk therapy to patients [7,39]. As a result, the quality of psychotherapeutic services that are provided range widely and may even be harmful [7]. Focus on overhauling and modernizing mental health education is urgently needed, including efforts to accredit programs and develop standards for didactic and clinical training.

Regarding psychiatry, recent efforts have been undertaken to improve and modernize medical education. The implementation of the WHO Mental Health Gap Action Programme-Intervention Guide (mhGAP-IG) in undergraduate and postgraduate levels of medical education seeks to improve knowledge and treatment of mental illnesses, with a long-term goal of strengthening the health system [40], [41], [42]. Broadly, the Cabinet of Ministers of Ukraine approved several resolutions in 2018 that require continuing medical education and ongoing professional development of healthcare workers, which may add stringency in regulation of psychiatry [43]. While we applaud these efforts, outside of the medical realm of treatment of mental illness, there is little in the way of regulation and training in modern mental health approaches.

Relatedly, there is a wide literature gap regarding mental health both in Ukraine and in the wider region; for example, Eastern European countries have the lowest publication rate of mental health research in Europe [3]. Further, multiple studies note that many commonly used mental health instruments have not been validated in the region [44], [45], [46], and there are very few studies that investigate psychotherapeutic interventions or mental health economics. This is of utmost concern: lack of research results in lack of knowledge of the effectiveness of mental health interventions; of the robustness of the psychometrics of psychiatric measures; and of the current epidemiology of psychiatric illness.

Even as the mental health system evolves, there is a clear need of having providers trained as scientist-practitioners who can inform best practices tailored to Ukraine's unique context and guide the development of scientific protocols. Each of the spheres noted, including care provision, education, and research, impact one another: lack of contemporary mental health education results in limited use of evidence-based practices, lack of highly trained mental health professionals limits scientific inquiry into best practices, lack of funding impacts access to mental health care that is available, and so forth. Strengthening the mental health care system will require investment into each interconnected pillar.

### Parallels and Distinctions between Ukraine and the Eastern European and Central Asian Region

Ukraine, as well as other countries in Eastern Europe and Central Asia, has a unique historical legacy and ongoing challenges with quality mental health care provision that merits greater attention from domestic and international stakeholders [3,4]. Though we highlighted Ukraine's specific mental health context, there are similarities across the region, including high rates of mental health and substance use disorders; [4] lack of mental health research [3,4]; future impact of COVID-19; and insufficient resources allocated to mental health services and reliance on hospitalization for treatment [4]. At the same time, we also note distinctions within the region; for example, the Central Asian countries (including Kazakhstan, Kyrgyzstan, Tajikistan, Turkmenistan and Uzbekistan) have recently been called “a blind spot within the blind spot” in terms of global mental health focus [13]. These issues point to the need for greater emphasis on Eastern Europe and Central Asia, and considerations of the unique context of each country, in order to fully advance the global mental health agenda.

## Call to Action and Future Directions

We suggest the following recommendations as important considerations across the Eastern European and Central Asian region. Key areas include funding and modernizing mental health education; licensing and regulation of all mental health professions; financing quality research that advances knowledge of culturally responsive mental health care; implementing and requiring evidence-based mental health services in government-funded clinics; and advancing mental health care reform.

To address these goals, we offer the following considerations. First, there is enormous potential for region-wide efforts to collaborate and share knowledge. For example, Pinchuk and colleagues (2021) described a partnership between Ukraine, Armenia, Georgia, and Kyrgyz Republic that focuses on working towards implementation of the mhGAP-IG and strengthening mental health education [41]. Similarly, other authors have noted the possibility of capitalizing on interest among Central Asian countries to develop a mental health workforce training institute [47]. Stemming from lessons learned during the COVID-19 pandemic, future efforts may also utilize digital learning to offer new pathways [48]. Learning from neighboring countries that have had recent success in working towards mental health care reform, such as Czechia, may also serve as a blueprint for countries in early stages and accelerate progress [49].

Second, a main difficulty in mental health research often revolves around financing, particularly achieving and sustaining funding [2]. Successful grant applications commonly require history of funding, as well demonstrated experience associated with the intricacies of grants management, which can often be a barrier for many Eastern European and Central Asian academic institutions. Opportunities for capacity building specifically related to research management are an important next step to better position Eastern European and Central Asian institutions for competitive funding. Another factor related to funding eligibility are perceptions about the development status of Eastern European mental health care systems. In large part, this perspective may stem from the inclusion of many countries in the region as either European Union Member States or Associated Countries, as well as designations as either high-income or upper-middle income economies [47,50]. However, as described above, these designations do not reflect the existing mental health care realities. Finally, successful grant applications commonly require a robust track record of scholarly productivity in English-speaking arenas, yet there is an enormous dearth of mental health literature from Eastern European and Central Asian countries. Unfortunately, emigration of promising early career researchers to American and Western European universities may compound this issue. Greater investment in training and retaining mental health academics is vital to close this gap.

Third, greater focus on mental health-related stigma, on mental health knowledge, and on inclusion of individuals with lived experience of mental illness remain important areas within ongoing mental health reforms [51]. This includes monitoring healthcare provider stigma on mental illness [51], as well as improved community education on mental health awareness and self-care. At the same time, greater attention to prevention (and mental well-being) is equally important as attention to treatment of mental illness [52].

We also emphasize the unique strengths across Ukraine and throughout Eastern Europe and Central Asia. Unlike many other LMICs that rely heavily on task shifting of mental health care to non-specialist workers [53], Eastern Europe and Central Asia have a relatively large number of mental health professionals, particularly psychiatrists and psychosocial care providers (i.e., psychologists, social workers) [54]. In order to bolster education that may not have previously offered adequate training, [7] post-graduate training courses may be a promising solution to capitalize on this potential workforce and greatly enhance current treatment as usual. We anticipate this would require collaboration between funders and government, with priority areas associated with mental health research and educational programs. Additionally, investment from governmental bodies to ensure coverage of mental health services, as well as to provide commitment to continuing systematic reforms in the mental health sphere, will be needed. This includes focusing on deinstitutionalization (while simultaneously financing continuous coverage of mental health services in psychiatric hospitals during the transition to community care) [30] and fostering equal representation of professional non-medical associations involved in forming mental health policy. Overall, we believe that increased investment from government, educational institutions, researchers, and funders, as well as collaboration with partners in the region and internationally, are key to meeting the needs outlined in this paper.

We acknowledge that “in the context of mental healthcare, all countries are ‘developing’ to some extent [55]”. From this perspective, the intent of this review is not to needlessly criticize current efforts but rather to frame today's challenges as a call to action: in our efforts to ensure mental health care access and equity worldwide, we must not forget, and must instead prioritize, mental health care and training in Eastern European and Central Asian countries. We believe that increased attention to mental health care will only be increasingly critical in the coming years.

## Declarations

*Acknowledgements:* The preparation of the present manuscript was supported by the Boston University Medical Campus-Massachusetts General Hospital Global Psychiatric Clinical Research Training Program (T32MH116140). The sponsors had no role in study design; in the collection, analysis and interpretation of data; in the writing of the articles; and in the decision to submit it for publication.

*Contributors:* Both authors contributed equally to this manuscript. KH and SB are responsible for study conceptualization and literature search. KH wrote the first draft of the manuscript with feedback from SB. KH and SB both contributed to the response and editing of later versions of the manuscript.

*Declaration of interests:* The authors declare that they have no competing interests.
